# A case of Rathke cleft cyst concomitant with sellar/suprasellar arachnoid cyst

**DOI:** 10.1007/s13760-016-0705-3

**Published:** 2016-10-12

**Authors:** Shun Yamamuro, Sodai Yoshimura, Hideki Oshima, Atsuo Yoshino

**Affiliations:** Department of Neurological Surgery, Nihon University School of Medicine, 30-1 Oyaguchi kamichou, Itabashi-ku, Tokyo, 173-8610 Japan

Dear Editor,

Sir, we report here a rare case of Rathke cleft cyst (RCC) resembling sellar/suprasellar arachnoid cyst (SAC) on magnetic resonance (MR) imaging.

A 75-year-old male was admitted to our hospital complaining of visual field disturbance. Laboratory evaluations including hormonal tests demonstrated no abnormalities. His consciousness level was clear and neurological examinations revealed no abnormalities except for bitemporal hemianopia. MR imaging disclosed a sellar/suprasellar cystic mass, which displayed low intensity on T1-weighted MR imaging, high intensity on T2-weighted MR imaging, and low intensity on fluid attenuated inversion recovery (FLAIR) imaging; no enhancement was evident after contrast medium administration (Fig. 1). A retracted pituitary and pituitary stalk were recognized at the bottom of the ballooning sella turcica with no waxy nodules or enhancement of the cyst wall (Fig. 1d, e). The lesion was diagnosed preoperatively as an SAC, not an RCC, because the intensity pattern of this cystic lesion was completely identical to that of cerebrospinal fluid (CSF).

**Fig. 1:**
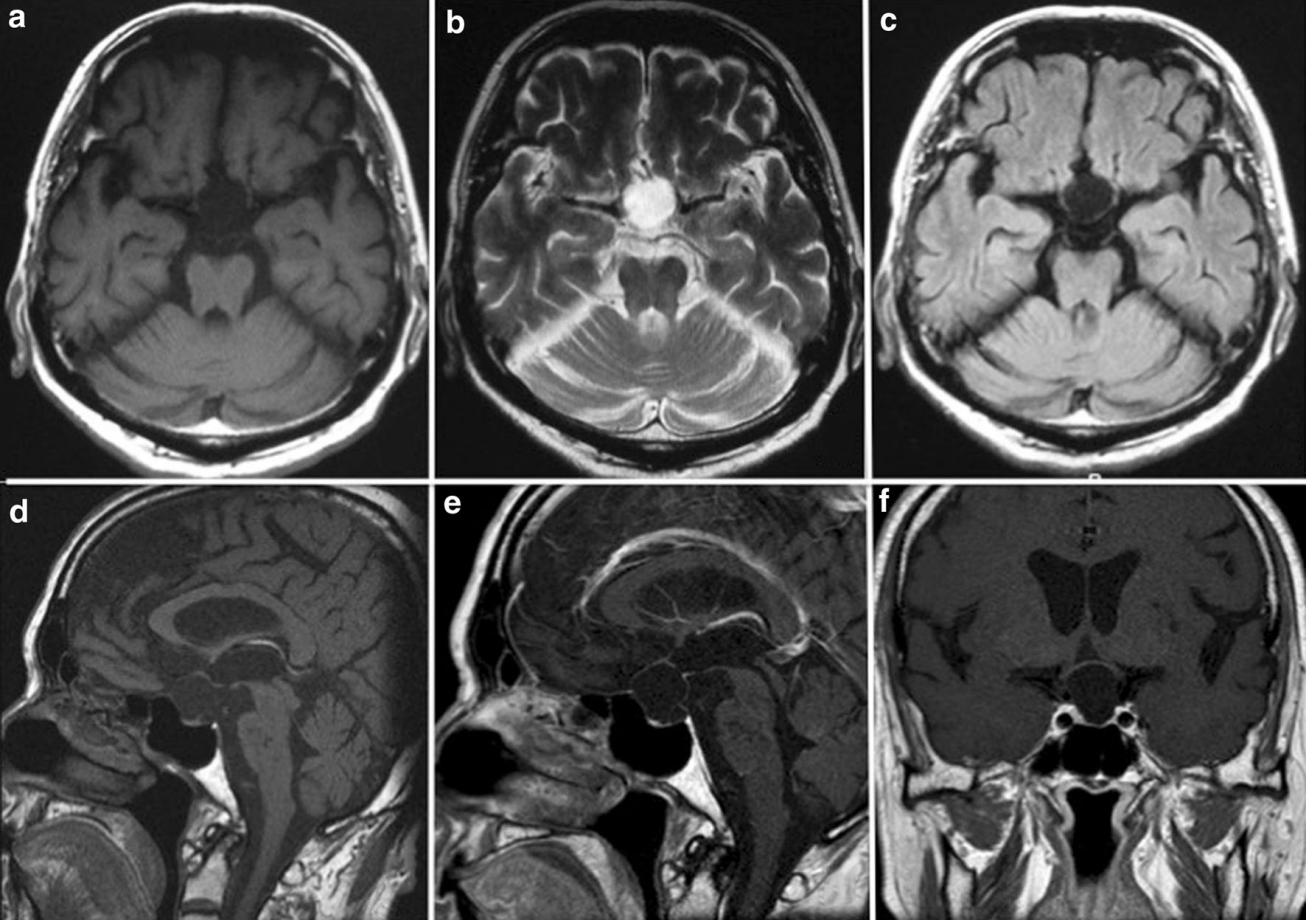
Preoperative MR imaging showing a cystic mass in the sellar region. The cystic contents displayed low intensity on T1-weighted MR imaging, high intensity on T2-weighted MR imaging, and low intensity on FLAIR imaging. No enhancement was evident after contrast medium administration (**a** axial T1-weighted MR imaging; **b** axial T2-weighted MR imaging; **c** axial FLAIR imaging; **d** sagittal T1-weighted MR imaging; **e** sagittal gadolinium-enhanced T1-weighted MR imaging; **f** coronal gadolinium-enhanced T1-weighted MR imaging)

The patient underwent simple cyst opening via endoscopic transnasal transsphenoidal surgery (TSS). The intraoperative findings indicated that the cystic contents were clear like CSF (Fig. 2a). The membrane component was partially excised for pathological diagnosis. Pathological examinations demonstrated that the membranous component consisted of cuboidal to low columnar epithelium, resting on fibrous tissue (Fig. 2b–d). These pathological findings were consistent with RCC. The patient’s postoperative course was uneventful. He did not experience major morbidity including postoperative rhinorrhea. His visual field disturbance improved after the operation. Postoperative computed tomography scans demonstrated reduction of the cyst volume (Fig. 2e, f).

**Fig. 2:**
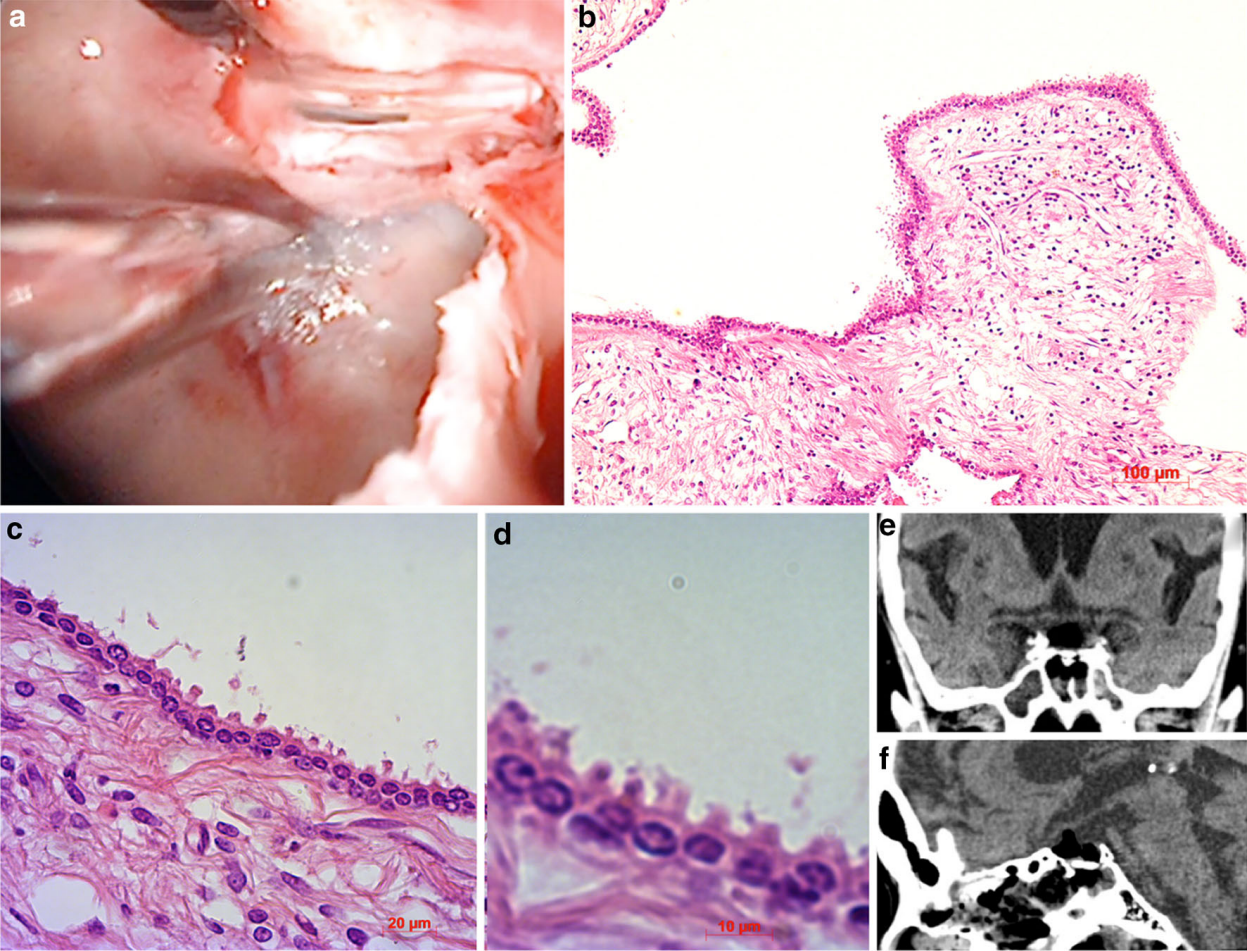
**a** Intraoperative photographs showing endoscopic views of the transnasal transsphenoidal surgery. Cystic contents that were clear like cerebrospinal fluid were recognized after dural opening. Photomicrograph of the cyst lining consisting of cuboidal to low columnar epithelial resting on fibrous tissue (**b** hematoxylin and eosin stain, original magnification ×100; **c** hematoxylin and eosin stain, original magnification ×400; **d** hematoxylin and eosin stain, original magnification ×1000). Immediate postoperative CT scans showing reduction of the cyst volume (**e** coronal: **f** sagittal)

The cystic contents of RCCs display various intensities on MR imaging depending on the nature of the cystic contents [1]. On the other hand, the cystic contents of SACs display the same intensity pattern as CSF. Rarely, when RCCs reveal only a cystic lesion containing clear, CSF-like fluid, with low intensity on T1-weighted MR imaging and high intensity on T2-weighted MR imaging, preoperative differentiation from SACs becomes difficult [2]. Although FLAIR imaging might be useful for discrimination in some cases, only a few reports have provided FLAIR imaging intensities for RCCs. The present case of RCC demonstrated the same intensity pattern as CSF on MR imaging, including FLAIR imaging, and this made discrimination difficult.

Different surgical techniques have been selected for RCCs and SACs [1, 3, 4]. Decompression and biopsy procedures are commonly performed for RCCs [1, 3]. On the other hand, cisternostomy, with fenestration of the cyst into the cisterns, is generally undertaken for SACs to prevent recurrence [4]. However, CSF leakage after cisternostomy via TSS still remains a troubling issue [5]. Furthermore, total resection of RCCs raises the risk of postoperative hypopituitarism [3]. Cisternostomy thus has a risk of over surgery, especially in the case of RCCs. Several recent reports have indicated the usefulness of simple cyst opening techniques for SACs via endoscopic transnasal TSS [5]. There are cases in which it is difficult to differentiate between RCCs and SACs. In those cases, simple cyst opening via TSS has been reported to be effective for both types of cysts, and we also obtained favorable outcome in our patient. Simple cyst opening via TSS may thus be an option for sellar/suprasellar lesions that on MR imaging present as a cystic mass with CSF-like intensity and where no certain differentiation between SAC and RCC is possible.

## References

[1] Nishioka H, Haraoka J, Izawa H, Ikeda Y (2006). Magnetic resonance imaging, clinical manifestations, and management of Rathke’s cleft cyst. Clin Endocrinol (Oxf).

[2] Mitsuhara T, Ikawa F, Ohbayashi N, Imada Y, Kazihara Y, Abiko S, Inagawa T (2005). Symptomatic Rathke’s cleft cyst mimicking suprasellar arachnoid cyst. No Shinkei Geka.

[3] Benveniste RJ, King WA, Walsh J, Lee JS, Naidich TP, Post KD (2004). Surgery for Rathke cleft cysts: technical considerations and outcomes. J Neurosurg.

[4] Dubuisson AS, Stevenaert A, Martin DH, Flandroy PP (2007). Intrasellar arachnoid cysts. Neurosurgery.

[5] McLaughlin N, Vandergrift A, Ditzel Filho LF, Shahalaie K, Eisenberg AA, Carrau RL, Cohan P, Kelley DF (2012). Endonasal management of sellar arachnoid cysts: simple cyst obliteration technique. J Neurosurg.

